# Aetiologies of Ear Infections Among Patients Who Visited King Fahad Hospital in Al-Baha, Saudi Arabia

**DOI:** 10.7759/cureus.67885

**Published:** 2024-08-27

**Authors:** Hasan Alfahemi, Mohammed Alghamdi, Mujtaba A Fadlalla, Muhammad Halwani, Rabei M Elbadry, Mujahid K Alghamdi, Fahad S Alghamdi, Abdullah M Alghamdi, Talal A Sallam

**Affiliations:** 1 Department of Medical Microbiology, Faculty of Medicine, Al-Baha University, Al-Baha, SAU; 2 Unit of Otolaryngology, Department of Surgery, Faculty of Medicine, Al-Baha University, Al-Baha, SAU; 3 Department of Pathology, Faculty of Medicine, Al-Baha University, Al-Baha, SAU; 4 Department of Medicine and Surgery, Faculty of Medicine, Al-Baha University, Al-Baha, SAU

**Keywords:** coagulase-negative staphylococcus, enterobacteriaceae, pseudomonas aeruginosa, otitis media, staphylococcus aureus, al-baha, ear infections, otitis externa

## Abstract

Background

Ear infections encompass otitis media (OM) which is a significant cause of hearing loss and otitis externa (OE) which may affect the surrounding tissues leading to serious complications. This study reports the common pathogens causing ear infections.

Methods

Microbiological, clinical, and demographic data of ear-infected patients who visited King Fahad Hospital in Al-Baha, Saudi Arabia, during the period from January 2019 to June 2023 were enrolled in this study.

Result

This study enrolled 307 patients aged 1-94 years, with a median age of 40 years (IQR=22-57). Overall, the detectable infection rate was 81.1% (n=249), while 18.9% (n=58) had no identified aetiology. Of all isolates, 178 (58%) were bacterial, while 71 (23.1%) were fungal. *Staphylococcus aureus* (*S. aureus*) followed by *Pseudomonas aeruginosa* (*P. aeruginosa*), *Enterobacteriaceae*, andcoagulase-negative *Staphylococcus* (CoNS) were the main bacterial isolates. Of the total 63 *S. aureus* isolates, 21 (33%) were methicillin-resistant (MRSA). A cohort of 227 subjects were diagnosed with either OM (n=178; 79.5%), OE (n=46; 20.5%), or both OM and OE (n=3; 1%). Of those with OM, children constituted 89.1% (41/46) as compared to 75.3% (134/178) of adults (p=0.041). The main isolates from OM patients were *S. aureus* followed by *P. aeruginosa *and fungi. Of 49 OE patients, 16 (32.7%) had no identified pathogen, while 15 (30.6%) had fungi, and 13 (29.5%) had *P. aeruginosa.*

Conclusions

Ear infections in general were mainly bacterial followed by fungal with a considerable proportion of unidentified aetiology. A significant proportion of *S. aureus* isolates were MRSA. *S. aureus *followed by* P. aeruginosa* and fungi were the main causes of OM, while fungi followed by *P. aeruginosa* were the main causes of OE.

## Introduction

Infection of the ear encompassing otitis media (OM) and otitis externa (OE) can be serious owing to the complications that may occur as a result. Around 1.23 billion people are affected by OM, ranking the fifth global burden of disease and the second cause of hearing loss [1,2]. It mainly affects children and is the main impetus for antibiotic prescription for children by ear, nose, and throat (ENT) specialists [1,3]. In Saudi Arabia, a prevalence of otitis media with effusion (OME) of 7.5% among eight-year-old children has been reported [4,5], and around 80% of children have suffered at least one episode of acute otitis media (AOM) with 80-90% having experienced at least one episode of OME prior to school entry age [6]. Acute otitis externa (AOE), also known as swimmer’s ear, is very prevalent among young children and swimmers, especially during summer and in warm, humid climates. Apart from the elderly, AOE has been reported in all age groups, more frequently in females than males [7], and is recognized as one of the top 10 reasons for antibiotic prescriptions in general practice [8]. In Saudi Arabia, OE has been reported as a major ear infection that mainly affects males over 40 years of age [9].

In different geographical locations of the world, reports varied in the aetiology and prevalence of ear infections. Varying bacterial species have been reported as the main cause of AOM and OME [10]. Several studies reported different bacterial species that were isolated from patients with OM [11-15], while bacteria and fungi were the main isolated pathogens from cases of OE [16-18]. In Saudi Arabia, bacteria and fungi have been reported as the main pathogens causing ear infections in general [19] and OM [20] and OE [9] in particular.

Ear pathogens in terms of type and magnitude seem to vary globally and within Saudi Arabia. Consequently, the pattern of ear infection in the Al-Baha region may also differ in terms of the most common aetiologies and prevalence. Therefore, the status of ear infections in various regions of the country needs to be evaluated. This in turn will contribute towards establishing the big picture of the problem of ear infection in the country at large and towards launching a suitable ear infection treatment stewardship which varies based on the specific aetiology. Therefore, this study aims to investigate the common aetiological agents of ear infections in the Al-Baha region.

## Materials and methods

Study area

The Al-Baha region lies in the southwest of the Kingdom of Saudi Arabia, between the holy city of Makkah and Aseer region. It has a population of 461,360 people and an area of 10,362 km2 [21]. The region encompasses high mountainous provinces and low coastal land provinces in Tehama with weather variation between cold winter and balmy summer in the mountains to mild winter and hot summer in the coastal provinces.

Study design, setting, and subjects

This is a retrospective cross-sectional study which included ear swab culture results of patients who visited the King Fahad Hospital in Al-Baha, Saudi Arabia, with signs and symptoms of ear infections. The hospital is designated as a tertiary hospital by the Ministry of Health (MOH). It is one of the largest hospitals in the Al-Baha region, with a capacity of over 400 beds. It serves the people of the seven governates of the Al-Baha region.

The study protocol was approved by the Ethics Research Committee (ERC) of the Faculty of Medicine, Al-Baha University (approval number: REC/MIC/BU-FM/2023/10R). Data were obtained with a written consent from the King Fahad Hospital.

All ear swabs which were collected consecutively during the study period from January 2019 to June 2023 were included in this study. These consisted of 369 ear swabs that were collected consecutively from 307 patients who presented with otalgia and/or ear discharge and who were referred for microbiological investigation. This sample size has a confidence level of over 99% and a margin of error of less than 1%. Sterile ear swabs were used to swab infected ears to collect sample material under aseptic conditions.

Laboratory investigation

The swabs were subjected to wet preparation and Gram-staining to check the presence of potential bacterial pathogens and fungi. Swabs were also cultured on chocolate agar incubated in 5-10% CO2 and 5% sheep blood agar and thioglycolate broth incubated aerobically at 37°C. Sabouraud dextrose agar was used for fungal cultures which were incubated at 30°C. Identification of the bacterial isolates was performed using the VITEK-2 computational system (bioMérieux, France) which is an automated microbiology system that uses growth‑based technology involving colorimetric reagent cards incubated and interpreted automatically [22]. The VITEK-2 compact system helps in identifying bacterial and fungal isolates and their response to various antimicrobials. This system was evaluated, and it was 95.8% compatible with the reference API strips (bioMérieux, France) in identifying Gram-positive cocci, Gram-negative rods, and yeast. Also, its accuracy was enhanced to reach 98.3% by the addition of confirmatory tests [22].

Quality control

All results of this study were obtained under the quality control program which was used by the Microbiology Section of the Laboratory and Blood Bank Department of King Fahad Hospital. All culture and identification procedures were performed in accordance with the Clinical and Laboratory Standards Institute (CLSI) guidelines M 100 S 29 and the Central Board for Accreditation of Healthcare Institutes (CBAHI) guidelines to assure the accuracy of results. The CLSI guidelines provide precise information and detailed instructions for microbial culturing along with the assessment of the microbial test precision and accuracy. The CBAHI guidelines ensure that all equipment used in the microbiology laboratory are appropriately calibrated and maintained and all microbial culture procedures are conducted safely.

Statistical analysis

Microbiological, clinical, and demographic data of patients were coded, entered, and analyzed using IBM SPSS Statistics for Windows, Version 25.0 (Released 2017; IBM Corp., Armonk, New York, United States). Quantitative analysis, mean, and standard deviation were used to express continuous data, while absolute and relative frequencies were used to express categorical variables. The chi-squared test was used to test the association between the dependent and independent variables. A p-value of <0.05 was considered statistically significant.

## Results

Of the 307 patients, 151 (49.2%) were males, and 156 (50.8%) were females. Their ages ranged from one to 94 years, with a median of 40 years (IQR=22-57). The patients consisted of three age groups encompassing 64 (20.8%) children aged 1-18 years, 89 (29%) adults aged 19-39 years, and 154 (50.2%) older adults aged ≥40 years (Table 1). Overall, the detectable rate of infection was 81.1% (249 of 307) with an infection rate that did not significantly differ between males and females, but increased, albeit insignificantly, with the progress of age (Table 1).

**Table 1:** Demographic characteristics of patients with ear infections and their association with the rates of infection (n=307).

Variables	Categories	Positive	Negative	Total	P-value
		n (%)	n (%)	n (%)	
Sex	Male	125 (82.7)	26 (17.3)	151 (49.2)	0.296
	Female	124 (79.5)	32 (20.4)	156 (50.8)	
Age	1-18 years	49 (76.6)	15 (23.4)	64 (20.8)	0.060
	19-39 years	67 (75.3)	22 (24.7)	89 (29)	
	≥40 years	133 (86.4)	21 (13.6)	154 (50.2)	

The majority of patients (216/307; 70.4%) had one infection episode with a single pathogen, while 33 (10.7%) had multiple infection episodes with at least one pathogen. As compared to those with a single infection episode, rates of subjects with multiple infection episodes did not significantly differ (p>0.05) between children and adults or males and females.

Of all patients, 178 (57.9%) had bacterial pathogens, of which *S. aureus* was the main isolate followed by *P. aeruginosa*, *Enterobacteriaceae* (including *Escherichia coli*, *Klebsiella*, and *Enterobacter*), coagulase-negative *Staphylococcus *(CoNS), less common bacterial isolates, and mixed bacterial isolates and fungal isolates including *Aspergillus *species and *Candida *species. The less common bacterial isolates (n=16) consisted of six (37.5%) *Haemophilus parainfluenzae* (*H. parainfluenzae*), six (37.5%) *Streptococcus* spp., three (18.8%) *Corynebacterium* spp., and one (6.3%) *Stenotrophomonas maltophilia* (*S. maltophilia*) isolates. Of all isolates (n=249), 178 (71.5%) were bacterial pathogens, while 71 (28.5%) were fungal pathogens, of which 21 (29.6%) were *Candida* species and 29 (40.8%) were *Aspergillus *species. Rates of infection with almost all pathogens increased significantly (p=0.0001) with increasing age (Table 2). Of the total 63 *S. aureus* isolates, 21 (33%) were methicillin-resistant (MRSA).

**Table 2:** Rates of various pathogens isolated from different age groups of subjects with ear infections. p=0.0001. A p-value of <0.05 was considered statistically significant. Total* calculated isolates=249. **: *Escherichia coli, Klebsiella, *and *Enterobacter.*

Isolate	Age (years)			Total
	1-18	19-39	≥40	
	n (%)	n (%)	n (%)	n (%)*
Staphylococcus aureus	15 (23.8)	17 (27)	31 (49.2)	63 (25.3)
Pseudomonas aeruginosa	11 (19)	17 (29.3)	30 (51.7)	58 (23.3)
Enterobacteriaceae**	1 (6.7)	3 (20)	11 (73.3)	15 (6.4)
Coagulase-negative *Staphylococcus*	4 (25)	4 (25)	8 (50)	16 (6)
Less common bacterial isolates	9 (56.2)	3 (18.8)	4 (25)	16 (6.4)
Mixed growth	2 (18.2)	8 (72.7)	1 (9.1)	11 (3.6)
Fungi	7 (10)	15 (21.4)	48 (68.6)	70 (23.1)
Total	49 (19.8)	67 (26.9)	133 (53.4)	249 (100)

A cohort of 227 received full clinical diagnosis. Of these, 178 patients (57.9%) were diagnosed with OM, 46 patients (14.9%) were diagnosed with OE, and three patients (1%) were diagnosed with both OM and OE, while 80 patients (26.5%) did not receive a conclusive clinical diagnosis. Of the 224 subjects diagnosed with either OM or OE, the prevalence of OM among children aged 1-18 years (41/46; 89.1%) was significantly (p=0.041) higher than among adults (134/178; 75.3%).

The isolates significantly (p<0.001) associated with OM were *S. aureus* (28.9%) followed by *P. aeruginosa* and various fungi in addition to other less prevalent pathogens including *Enterobacteriaceae*, CoNS, and a diverse array of bacterial species or mixed isolates (Table 3). Conversely, the majority (34.1%) of patients diagnosed with OE did not have an identified pathogen, although fungi (30.6%) and *P. aeruginosa* (29.5%) were the most significant (p<0.001) organisms associated with this ear condition (Table 3). Of the three patients diagnosed with both OM and OE, the main microbial isolates identified were *P. aeruginosa*, fungi, and CoNS.

**Table 3:** Types of organisms causing different clinical presentations of ear infections. p<0.001. A p-value of <0.05 was considered statistically significant. OM: otitis media; OE: otitis externa; CoNS*: coagulase-negative *Staphylococcus*; OP*: other presentations.

Clinical presentation	Pathogens								
	S. aureus	P. aeruginosa	Enterobacteriaceae	CoNS*	Less common bacterial isolates	Mixed isolates	Fungi	No isolates	Total
	n (%)	n (%)	n (%)	n (%)	n (%)	n (%)	n (%)	n (%)	n (%)
OM	51 (29.1)	38 (22)	12 (6.9)	9 (5.2)	7 (4)	6 (3.5)	37 (21)	15 (8.6)	175 (100)
OE	1 (2)	13 (26.5)	0 (0)	3 (6.1)	0 (0)	1 (2.3)	15 (30.6)	16 (32.7)	49 (100)
OM and OE	0 (0%)	1 (33.3)	0 (0)	1 (33.3)	0 (0)	0 (0)	1 (33.3)	0 (0)	3 (100)
OP*	11 (13.8)	6 (6.9)	3 (3.4)	3 (3.8)	9 (10.3)	4 (4.6)	17 (22.8)	27 (33.8)	80 (100)
Total	63 (20.5)	58 (18.9)	15 (4.9)	16 (5.2)	16 (5.2)	11 (3.6)	70 (22.8)	58 (18.9)	307 (100)

## Discussion

This is the first study to report on ear infections in the Al-Baha region. The present study revealed an ear infection rate of 81.1%. This is slightly higher than (76.7%) [13] or comparable to (80.4%) what has been reported elsewhere [23]. Additionally, the infection rates were comparable in both males and females (82.7% and 79.5%, respectively) although higher rates among males (63.1% [13] and 92.7% [23]) have been reported by other investigators. The infection rate in the current study increased, albeit insignificantly, with the progress of age suggesting an old age-associated risk. Data on risks associated with ear infections among adults is scarce. However, Eustachian tube dysfunction or obstruction and an abnormality of the respiratory mucosal membrane lining the Eustachian tube, the middle ear space, and the mastoid air cells which constitute immune defensive barriers have been reported as possible risks associated with ear infection [24]. Moreover, poor healthcare, low standard of living conditions, and poor nutritional status have been suggested to play a role in the impairment of immune defense and consequently development of chronic suppurative otitis media (CSOM) [25]. Epidemiological data of the patients in this study were not available for risk factor analysis. However, increasing ear infection rate with increasing age is likely to be a direct outcome of old age-associated comorbidities and waning immunity which in turn may predispose to ear infections or to upper respiratory tract viral infection that may become complicated by secondary bacterial ear infections. Less efficient immune systems and other comorbidities such as diabetes mellitus and hypertension have been suggested to increase the chances of ear infection incidences among old-aged patients [26,27].

Of all isolates in this study, 71.4% were bacteria while 23.1% were fungi. A higher rate of bacterial infections (96.9%) and a considerably lower rate of fungal infections (2.4%) of the ear have previously been reported [23]. In the current study, the major bacterial isolates were *S. aureus* which encompassed 20.5% followed by *P. aeruginosa* which formed 18.9% and *Enterobacteriaceae* and CoNS each constituting 4.9% of all isolates. Elsewhere in Saudi Arabia, of all ear isolates reported, 20.7% were *P. aeruginosa*, 17.2% were yeast, 15.8%, were *S. aureus*, 12.4% were CoNS, and 8.2% were *P. mirabilis* [9], while *S. aureus* (54%) and *P. aeruginosa* (13%), with a lower rate (3%) of *S. pyogenes*, *Bacillus subtilis*, and *Proteus vulgaris*, have been also reported [19]. Reports from other countries have shown that ear isolates consisted of 27.3% *S. aureus*, 24.2% P. aeruginosa, 19.3% *Enterobacteriaceae*, and 12.6% fungi [28], while 21.2% and 8.9% of all isolates were *S. aureus* and *P. aeruginosa*, respectively [13]. The likely reason for this variation is the different study populations, in particular the age range and rates of males and females enrolled in addition to the cultural difference of the subjects included in these studies. The current study involved a larger sample size and a wider age range which may have accounted for the difference in the infection rate.

Consistent with other reports [11,5,9] that have established OM as a pediatric infection, the current study has demonstrated a significant association (p=0.041) of OM with those aged ≤18 years among the cohort of our subjects (n=224) who were diagnosed with OM or OE. In the current study, the prime organisms isolated from patients with OM were *S. aureus* (29.1%) followed by *P. aeruginosa* (22%), a finding that is in line with a previous report in Saudi Arabia where *S. aureus* was isolated from 25.6% followed by *P. aeruginosa* from 20.9% and CoNS from 18.6% of the isolates [9]. However, reports of the main pathogens of OM from other parts of the world were variable. One study reported *P. aeruginosa *(38%) followed by *S. aureus* (22%) as the main isolates [29]. Another study reported that up to 27% of the isolates from OM patients were *S. aureus*, while up to 25% were *P. aeruginosa* [30]. It has also been reported that *Proteus* spp., *S. aureus*, and *Pseudomonas* spp. constituted 26.5%, 24.6%, and 18% of all isolates, respectively [11]. Additionally, *S. aureus* (28.4%) followed by *P. mirabilis* (24.1%) and *P. aeruginosa* (16.7%) have also been reported [12]. The similarity of our findings to those from elsewhere in Saudi Arabia is attributable to the similar sample size and age range, although the male-to-female ratios were different, in addition to the similar geographical, climatic, and cultural influences. The differences in our findings from those reported in other parts of the world were likely to be due to geographical, climatic, and cultural differences rather than to the sample size, age range, or male-to-female ratio which are comparable to those in the current study.

The current study demonstrates that the highest proportion of the OE cases was caused by an unidentified aetiology (32.7%) as these cases were culture-negative, followed by fungi (30.6%) and *P. aeruginosa* (26.5%). This is inconsistent with the previous report in Saudi Arabia where yeast constituted 42.3%, CoNS consisted of 23.1%, and *S. aureus* and *Pseudomonas* each comprised 15.4% of the OE isolates despite the similarity in the sample size and age range [9]. However, the obvious difference is the male-to-female proportions as our OE cohort consisted of close proportions of males and females compared to 80% females in the previous study [9]. The current findings also differ from those reported in Jordan where each of *P. aeruginosa* and CoNS comprised 22%, fungi encompassed 17%, and *S. aureus* constituted 10.9% of the isolates [16]. Additionally, other studies reported fungi (38.7%) followed by *P. aeruginosa* (19.3%), *S. epidermidis* (16.1%), *Klebsiella* spp. (9.7%), and *S. aureus* (9.6%) as the most frequently isolated organisms among cases of OE [18], whereas *P. aeruginosa* (24.8%) followed by CoNS (17.6%) and *S. aureus* (11.2%) as the major isolates were also reported [31]. All these studies differ in their study populations in terms of sample size, age range, and male-to-female ratios which seem to be the likely reasons for their different findings compared to those of the current study.

Further, the variation in infection rate and aetiological organisms causing either OM or OE between the current study and the previous reports in other parts of the globe is likely to reflect variation in the clinical condition of the infected ear whether AOM, ASOM, or OME. In 50-90% of patients with AOM and OME, *S. pneumoniae*, nontypable *H. influenzae*, and *M. catarrhalis* were the most frequently isolated pathogens [10], while other studies reported *P. aeruginosa* followed by *S. aureus* as the most common bacterial isolates among patients with CSOM [32]. In fact, the present study included a cohort of patients who were diagnosed with OM and OE with no further clinical categorization. Furthermore, reasons for variation in infection rate and aetiology could also reflect differences in socioeconomic status, geographical location, climatic factors, and racial as well as individual factors related to anatomical and structural ear defects. This variation is not unique to this study as variations have also been noted across various studies. Further investigations that reveal the possible role of these factors in variable aetiology and infection rates are needed.

The adult subjects in this study have almost equally high infection rates compared to those of children whose anatomical differences predispose them to higher infection rates. The wide age range may appear to have influenced the findings of this study. However, the children's sample size with a confidence level of 99% and a margin of error of less than 1% in this study was 64 matching well the actual number of children included in the study. Thus, they were adequately represented in the total sample size. The almost equally high rate of infections among both children and adults could be related to the retrospective nature of the study in which errors due to confounding bias may exist. The high rate of adults with ear infections could be related to the chronic nature of ear infections and to the clinical setting where this study was conducted making adults overrepresented. However, a larger sample size could possibly better represent the various age groups and thus compensates for any possible bias although a prospective study is indispensable to further verify this point.

In the current study, the less frequently isolated pathogens which included *H. parainfluenzae*, *Corynebacterium* spp., *Streptococcus* spp., and *Stenotrophomonas maltophilia* were not reported in other studies probably due to technical reasons as the system of identification used in our studies was more advanced and efficient. The other reason is the possibility that these organisms are not part of the aetiology of ear infections in some other regions due to socioeconomic, geographical, and climatic factors. Additionally, that *S. pneumoniae* was less frequently isolated in addition to the complete absence of *H. influenzae* among our subjects is likely related to the possibility that our subjects have developed immunity against them because of vaccination against both pathogens that have been included within the Saudi Extended Program on Immunization (EPI).

The possible aetiology of ear infection in the cases with negative ear swab culture could be either viral or possibly bacterial that failed to grow in culture due to prior antibiotic treatment. Viral aetiology of ear infection may not routinely be investigated in some healthcare settings and therefore diagnosed by exclusion. Viral OM commonly occurs as a complication of the common cold in children as rhinoviruses, respiratory syncytial virus (RSV), influenza virus, and adenovirus were detected in the middle ear fluids in 20-40% of cases of OME in children who were likely to have had Eustachian tube dysfunction [33]. Additionally, rhinoviruses and coronaviruses have been reported as the main cause of the common cold, and they can cause OM [34]. A high prevalence of symptomatic viral upper respiratory tract infections (URTI) among young children with >60% of cases complicated by AOM and/or OME has been reported [34].

The majority of our patients (216; 70.4%) had one infection episode with one pathogen; nevertheless, 33 (10.7%) had multiple infection episodes with at least one pathogen. The recurrence of ear infections among our subjects is not specifically known as no sufficient clinical data were available; however, this might occur due to various reasons. These include allergies, sinusitis, ear injuries, and bacterial infections from URTI [35]. Of all *S. aureus* isolates in the present study, 33% were MRSA which signals a possible hospital source of infection given the chronic nature of ear infections and consequently the need for repeated use of medical devices for diagnosis or treatment. However, the possibility of community-associated MRSA infections cannot be excluded. Inadequate clinical data for our patients hindered establishing the nature of the source of infections. However, the high rate of MRSA in the current study is consistent with rates of MRSA of 24-51% that have been reported in various regions of Saudi Arabia suggesting a growing threat of this pathogen [36]. Globally, the prevalence of MRSA causing various infections reached 60% in Europe, 80-100% in Africa, and up to 90% in America [37]. High prevalences of MRSA indicate a higher risk for patients and a need for second-line more toxic drug treatment which consequently raises costs and adverse effects and may drive further antimicrobial resistance [38]. Therefore, strict implementation of preventive measures including early diagnosis, isolation of infected patients, regular disinfection, and prophylactic intranasal mupirocin is vital for the effective prevention and management of MRSA infections [39].

Strength and limitations

The major strength of this study is that it provides essential data on ear infections in the Al-Baha region where ear infection studies are scarce. One limitation of this study was the lack of conclusive clinical diagnosis of all cases. This could have provided findings on the aetiology of the respective clinical condition of the ear and could have contributed towards the explanation of recurrences of ear infections among the study participants. The retrospective nature of the study and the subsequent selection biases are another limitation. The wide age range of participants may have influenced the findings of this study. Therefore, a larger sample size could possibly and adequately represent the various age groups and thus compensates for this possible bias.

## Conclusions

Ear infections in this study were mainly bacterial followed by fungal with a considerable proportion of no identified aetiology. A significant proportion of *S. aureus* ear isolates were MRSA. The main isolates were *S. aureus*, *P. aeruginosa*, and fungi being the main causes of OM, while fungi followed by *P. aeruginosa* were the principal causes of OE. Determining the common aetiologies of the different ear infections contributes towards establishing suitable ear infection treatment policies which vary based on the specific aetiology.
